# Incorporating multiple interventions in meta-analysis: an evaluation of the mixed treatment comparison with the adjusted indirect comparison

**DOI:** 10.1186/1745-6215-10-86

**Published:** 2009-09-21

**Authors:** Christopher O'Regan, Isabella Ghement, Oghenowede Eyawo, Gordon H Guyatt, Edward J Mills

**Affiliations:** 1 Department of Epidemiology, London School of Hygiene & Tropical Medicine, London, UK; 2 Department of Statistics, University of British Columbia, Vancouver, British Columbia, Canada; 3 Faculty of Health Sciences, Simon Fraser University, Burnaby, British Columbia, Canada; 4 Department of Clinical Epidemiology & Biostatistics, McMaster University, Hamilton, Ontario, Canada; 5 Faculty of Health Sciences, University of Ottawa, Ottawa, Ontario, Canada

## Abstract

**Background:**

Comparing the effectiveness of interventions is now a requirement for regulatory approval in several countries. It also aids in clinical and public health decision-making. However, in the absence of head-to-head randomized trials (RCTs), determining the relative effectiveness of interventions is challenging. Several methodological options are now available. We aimed to determine the comparative validity of the adjusted indirect comparisons of RCTs with the mixed treatment comparison approach.

**Methods:**

Using systematic searching, we identified all meta-analyses evaluating more than 3 interventions for a similar disease state with binary outcomes. We abstracted data on each clinical trial including population *n *and outcomes. We conducted fixed effects meta-analysis of each intervention versus mutual comparator and then applied the adjusted indirect comparison. We conducted a mixed treatment meta-analysis on all trials and compared the point estimates and 95% confidence/credible intervals (CIs/CrIs) to determine important differences.

**Results:**

We included data from 7 reviews that met our inclusion criteria, allowing a total of 51 comparisons. According to the *a priori *consistency rule, we found 2 examples where the analytic comparisons were statistically significant using the mixed treatment comparison over the adjusted indirect comparisons and 1 example where this was vice versa. We found 6 examples where the direction of effect differed according to the indirect comparison method chosen and we found 9 examples where the confidence intervals were importantly different between approaches.

**Conclusion:**

In most analyses, the adjusted indirect comparison yields estimates of relative effectiveness equal to the mixed treatment comparison. In less complex indirect comparisons, where all studies share a mutual comparator, both approaches yield similar benefits. As comparisons become more complex, the mixed treatment comparison may be favoured.

## Background

Acknowledging their enormous value for health intervention decision-making, clinicians, drug manufacturers, regulatory agencies and the public are now requiring meta-analysis to identify the most effective intervention among a range of alternatives.[1] As meta-analysis grows in popularity, investigators have endeavoured to further enhance its usefulness by proposing extensions meant to accommodate a number of challenges. One important challenge is choosing from a number of potentially competing interventions, not all of which have been subject to direct comparison in properly conducted randomized trials; herein referred to as indirect comparisons.

Until recently, meta-analysis addressed indirect comparisons using flawed methods that examined only intervention groups and ignored control event rates.[2] In the last decades, methodological advances,[3] most notably, the adjusted indirect comparison, first reported in 1997,[4] and the mixed treatment comparison, first reported in 2003, [5] have provided more sophisticated methods for quantitatively addressing indirect comparisons.

The adjusted indirect comparison, first reported by Bucher et al.,[4] enables one to construct an indirect estimate of the relative effect of two interventions A and B, by using information from randomized trials comparing each of these interventions against a common comparator C (e.g., placebo or standard treatment). In this approach, direct estimates of the relative effects of A versus C and B versus C, together with appropriate measures of uncertainty, are obtained using standard pairwise meta-analysis. These estimates are then appropriately combined to produce an indirect estimate of the relative effect of A versus B. A suitable measure of uncertainty for the indirect estimate is also produced.

The multiple treatment approaches, based on developing methods by several investigators,[6,7]most recently Lu and Ades,[8] is a generalization of standard pairwise meta-analysis for A versus B trials, to data structures that include, for example, A versus B, B versus C, and A versus C trials. This approach, which can only be applied to connected networks of randomised trials, has two important roles: (1) strengthening inference concerning the relative efficacy of two treatments, by including both direct and indirect comparisons of these treatments, and (2) facilitating simultaneous inference regarding all treatments, in order to simultaneously compare, or even rank, these treatments.[8]

The adjusted indirect comparison and the mixed treatment comparison approach can be implemented through a range of methods, including frequentist, Bayesian and various subspecies of each.[9]

The basic assumptions underlying the adjusted indirect comparison and mixed treatment comparison approaches are similar to but more complex than the assumptions concerning the standard meta-analysis approach. Just like standard meta-analysis, both approaches rely on the homogeneity assumption, which states that trials are sufficiently homogeneous to be quantitatively combined. In addition, both approaches require a similarity assumption - namely, that trials are similar for moderators of relative treatment effect. The mixed treatment comparison approach also requires a consistency assumption, which is needed to quantitatively combine direct and indirect evidence.[10]

Both adjusted indirect comparison and mixed treatment comparison approaches to evaluating the relative impact of multiple alternative treatments have strengths and weaknesses.[11] The multiple treatment comparison uses both direct and indirect evidence. The adjusted indirect method is comparatively simple and interpretable by users, but requires that an intervention can only be compared with another intervention when they share a mutual comparator (eg. placebo).[4] The mixed treatment comparison may be less intuitive as it can permit comparisons when interventions do not share a comparator as it creates a conceptual network[12,13] as well as borrows power from trials that were not available for use in the adjusted indirect comparison approach.[14]

Meta-analysts, agencies, and readers are now attempting to gain further insight into the relative merits of the two approaches.[15] New US government initiatives to determine the comparative effectiveness of interventions require the use of indirect evidence, but do not provide guidance on what approach to use. Others, such as UK's National Institute for Clinical Excellence (NICE) provide advice on the particular use of mixed treatment comparisons and adjusted indirect comparisons.[15] To further elucidate the relative performance of the adjusted indirect comparison and mixed treatment comparison methods, we applied both approaches to different comparative studies that evaluated the effectiveness of multiple competing treatments for diverse health conditions. Our objective is to determine whether the adjusted indirect comparison approach generates results comparable to those produced by the mixed treatment comparison approach. We aim to determine if there are circumstances where one method is preferable.

## Methods

### Eligibility Criteria

We included systematic reviews of randomized clinical trials involving at least 4 different treatments (i.e., health interventions used for treatment or prevention of the same medical condition), as networks of three health interventions have already received considerable study.[2,3,16] If a treatment consisted of several doses, we considered all doses to be equivalent. We also considered no-treatment and placebo to be equivalent. Whenever present, we excluded cluster randomized trials from these systematic review along with crossover trials and trials reporting only continuous outcomes.

### Search Strategy

We (EM, OE) searched independently, in duplicate, PubMed from inception to January 2008 using the following search strategy: "network AND meta-analysis," "mixed treatment AND meta-analysis," "indirect comparison," "indirect AND meta-analysis," and "mixed treatment AND meta-analysis." Our search was limited to English-language articles. We supplemented our search strategy and findings from a review of network geometry of studies[13] and from our own meta-analyses of multiple treatments (Perri D, O'Regan C, Cooper C, Nachega JB, Wu P, Tleyjeh I, Philips P, Mills EJ: **Antifungal treatment for systemtic candida infectons: A mixed treatment comparison meta-analysis**. Unpublished).[17]

### Data Abstraction

We (EM, OE) abstracted independently, in duplicate, information addressing the systematic review aims, number of trials per comparison, number of individuals with each specific outcome and number of individuals randomised to each intervention.

### Statistical analyses

We first plotted the geometric networks of comparisons to graphically display what indirect comparisons our analyses aimed to assess.

We conducted the mixed treatment comparisons using fixed effects models similar to those introduced by Lu and Ades.[8] Although several definitions exist, we interpret that the fixed effects approach assumes that there is a single true value underlying all the study results. That is, those studies would yield similar effects regardless of the particular population enrolled, the intervention chosen, and the strategy for measuring the outcome of interest. A fixed effect model aims to estimate the common-truth effect and the uncertainty around this estimate.[18] We considered separate models for each outcome category (i.e., mortality, response) using approximately non-informed priors. We used these models as a basis for deriving the odds ratio [OR] for each treatment comparison with 95% Credible Intervals (CrIs) - the Bayesian equivalent of a classical confidence interval.

We estimated the posterior densities for all unknown model parameters using MCMC (Markov chain Monte Carlo) simulation, as implemented in the software package WinBUGS Version 1.4. Specifically, we simulated two MCMC chains starting from different initial values of select unknown parameters. Each chain contained 20,000 burn-in iterations followed by 20,000 update iterations. We assessed convergence by visualizing the histories of the chains against the iteration number; overlapping histories, that appeared to mix with each other, provided an indication of convergence. We based our inferences on the (convergence) posterior distributions of the relevant parameters. In particular, we estimated the OR for a given treatment comparison by exponentiating the mean of the posterior distribution of the log OR, and constructed the corresponding 95% CrI by exponentiating the 2.5^th ^and 97.5^th ^percentiles of the posterior distribution of the log OR. Other parameters were estimated as means of corresponding posterior distributions.

We measured the goodness of fit of our models to the data by calculating the residual deviance. Residual deviance was defined as the difference between the deviance for the fitted model and the deviance for the saturated model, where the deviance uses the likelihood function to measure the fit of the model to the data. Under the null hypothesis that the model provides an adequate fit to the data, the residual deviance is expected to have a mean equal to the number of unconstrained data points.

For our relative effect sizes used in the adjusted indirect comparison analyses, we used the same data as for the mixed treatment comparison analyses. We conducted multiple meta-analyses of head-to-head comparisons to obtain ORs and 95% Confidence Intervals [95% CIs]. As with the mixed treatment analyses, we applied the fixed effects method. Once we obtained the summary estimates of pooled head-to-head evaluations with CIs, we applied the adjusted indirect comparison approach.[4]

For each systematic review, we determined if there were important inconsistencies between the adjusted indirect comparison and mixed treatment comparison approaches by comparing the 95% CrI produced by the former approach against the 95% CI produced by the latter approach for the OR of each feasible treatment comparison. We diagnosed inconsistency by assessing departures from an *a priori *determined consistency rule stating that the lower and upper endpoints of the two types of intervals should not differ by more than 0.25 and 0.75, respectively, and the estimated ORs should not differ by more than 0.5. EM and IG performed all statistical analyses.

## Results

**Figure 1:**
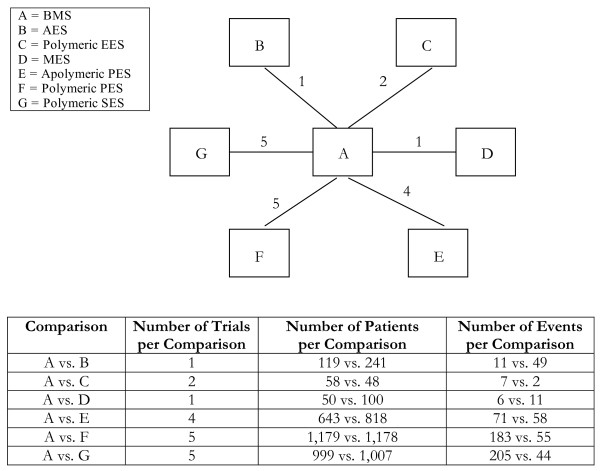
**Star-network of evidence formed by the seven stent treatments on target lesion revascularization event rates, together with information on the number of trials, number of patients and number of events per (direct) treatment comparison**. Each treatment is a node in the network. The links between nodes are trials or pairs of trial arms. The numbers along the link lines indicate the number of trials or pairs of trial arms for that link in the network.

**Figure 2:**
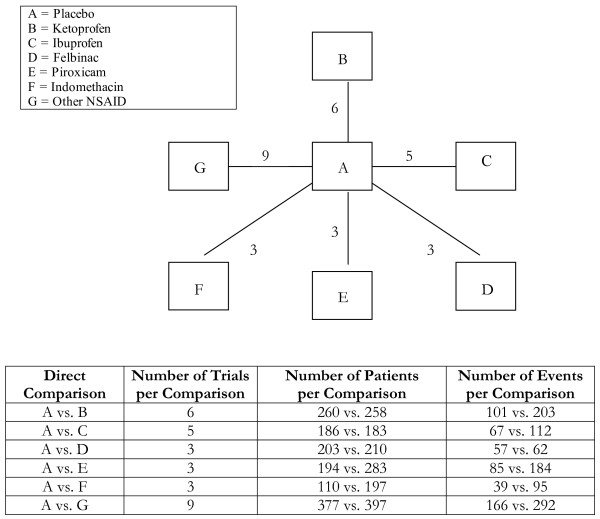
**Star-network of evidence formed by the treatments Placebo, Ketoprofen, Ibuprofen, Felbinac, Piroxicam, Indomethacin and Other NSAID, together with information on the number of trials, number of patients and number of events per (direct) comparison**.

**Figure 3:**
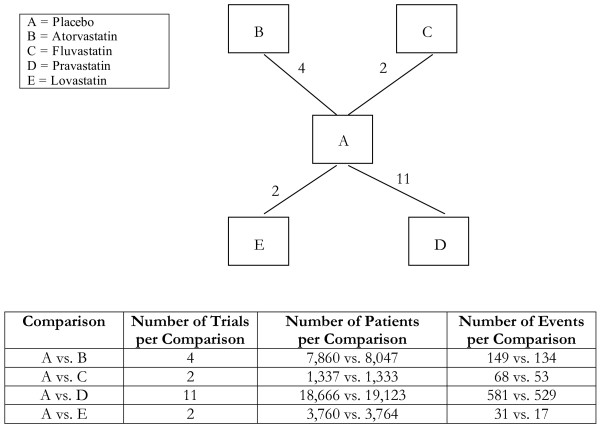
**Star-network of evidence formed by the four statin treatments and the placebo treatment in primary prevention of cardiovascular mortality, together with information on the number of trials, number of patients and number of events per (direct) comparison**.

**Figure 4:**
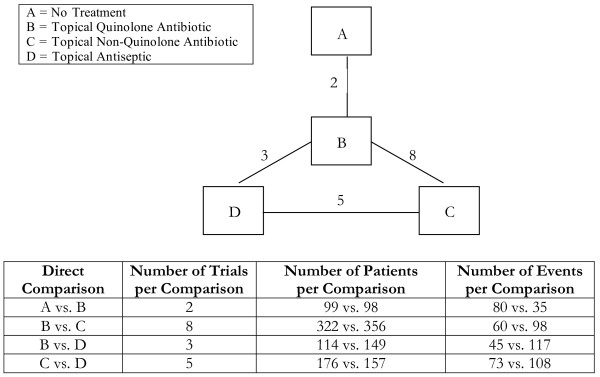
**Single-loop network of evidence formed by the four antibiotic and antiseptic treatments, together with information on the number of trials, number of patients and number of events per (direct) treatment comparison**.

**Figure 5:**
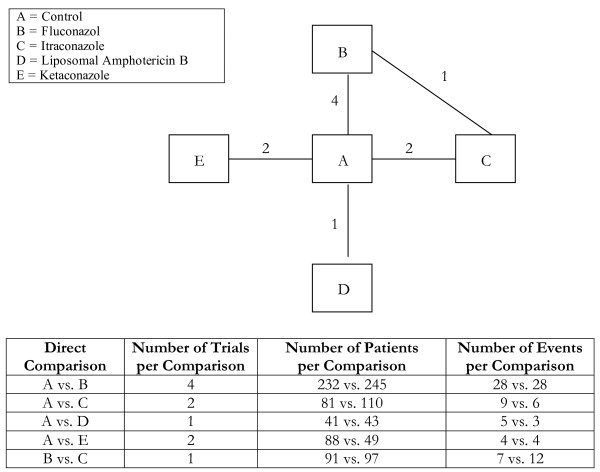
**Single-loop network of evidence formed by five antifungal treatments, together with information on the number of trials, number of patients and number of events per (direct) treatment comparison**.

**Figure 6:**
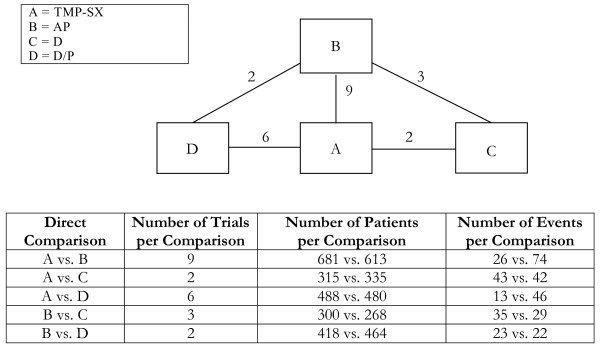
**Multi-loop network of evidence formed by the four treatments for prevention of Pneumocystis carinii pneumonia, together with information on the number of trials, number of patients and number of events per (direct) treatment comparison**.

**Figure 7:**
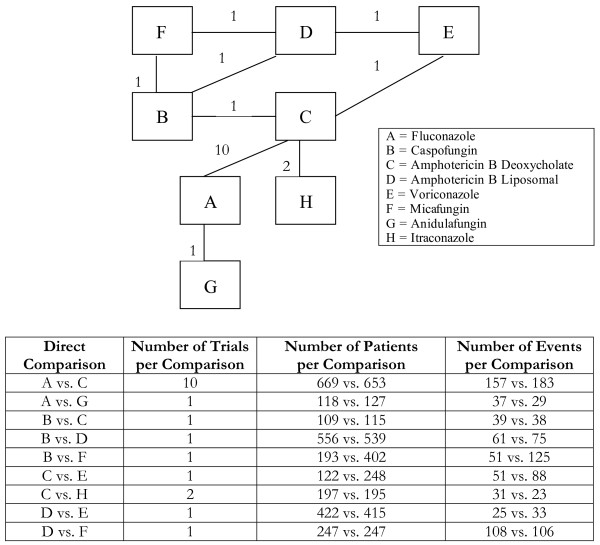
**Multi-loop network of evidence formed by the eight antifungal treatments, together with information on the number of trials, number of patients and number of events per (direct) treatment comparison**.

We identified 44 potentially relevant systematic reviews of the effectiveness of multiple treatments for different health conditions, including two of our own reviews that were ongoing during the search period (Perri D, O'Regan C, Cooper C, Nachega JB, Wu P, Tleyjeh I, Philips P, Mills EJ: **Antifungal treatment for systemtic candida infectons: A mixed treatment comparison meta-analysis**. Unpublished).[17] We narrowed down the scope of our search by excluding 13 reviews that incorporated fewer than 4 treatments, 9 reviews that excluded eligible data for comparisons, 3 reviews that did not create a network of comparisons, and 12 reviews that did not provide data on individual outcomes in each study. In total, we included seven systematic reviews in our analyses (Perri D, O'Regan C, Cooper C, Nachega JB, Wu P, Tleyjeh I, Philips P, Mills EJ: **Antifungal treatment for systemtic candida infectons: A mixed treatment comparison meta-analysis**. Unpublished) [4,17,19-22]with three different types of network structures: (I) star-network, having a common comparator and containing no loops (figures 1, 2, 3), (II) single-loop network (figures 4 and 5), containing only one loop, and (III) multi-loop network, containing two or more loops (figures 6 and 7). All seven reviews were published between the years 1997 to present.

### Number of comparisons

The seven systematic reviews retained in our analyses included between 4 and 8 treatments. Four reviews did not have a no-treatment control intervention (Perri D, O'Regan C, Cooper C, Nachega JB, Wu P, Tleyjeh I, Philips P, Mills EJ: **Antifungal treatment for systemtic candida infectons: A mixed treatment comparison meta-analysis**. Unpublished). [4,20,22] The number of trials included in the seven systematic reviews ranged from 10 to 29. Two reviews had insufficient mutual comparator arms to allow the adjusted indirect comparison evaluation on each intervention (Perri D, O'Regan C, Cooper C, Nachega JB, Wu P, Tleyjeh I, Philips P, Mills EJ: **Antifungal treatment for systemtic candida infectons: A mixed treatment comparison meta-analysis**. Unpublished). [21] There were no three or greater-armed trials found in any of the seven systematic reviews.

Analyses 1-3 (figures 1, 2, 3) represent star-shaped comparisons whereby each intervention shares a mutual comparator. Analysis 4 and 5 (figures 4 and 5) are networks with a single loop demonstrating that multiple interventions have been compared, but do not necessarily have a mutual comparator across treatment. Analyses 6 and 7 (figures 6 and 7) are multi-loop comparisons whereby more treatments exist that have not had mutual comparators.

#### Analysis 1. Drug-eluting stents compared to bare-metal stents on target lesion revascularization event rates[22]

We evaluated the impact of drug-eluting stents compared to bare-metal stents on the outcome of target lesion revascularization event rates on the basis of 18 2-arm randomised trials comparing 7 different treatments. Figure 1 displays the network of evidence available from these trials. Table 1 shows the results of the pairwise treatment comparisons when using direct, head-to-head data (in bold), the mixed treatment approach and the adjusted indirect comparison approach. In a single instance, the mixed treatment comparison approach found a significant difference between the effects of two treatments when the adjusted indirect comparison approach did not. According to the *a priori *consistency rule, the estimated ORs and associated uncertainty intervals were importantly different between the two approaches for only four pairwise treatment comparisons.

**Table 1:** Drug-eluting stents compared to bare-metal stents on revascularization status[22].

**Treatment Comparison**	**Mixed Treatment Comparison**		**Adjusted Indirect Comparison**	
	
	**Odds Ratio**	**95% Credible Interval**	**Odds Ratio**	**95% Confidence Interval**
AES vs. BMS	2.61	(1.32, 5.44)	**2.51**	**(1.22, 5.56)**
Polymeric EES vs. BMS	0.31	(0.04, 1.63)	**0.37**	**(0.06, 2.14)**
MES vs. BMS	0.93	(0.32, 2.88)	**0.91**	**(0.28, 3.19)**
Apolymeric PES vs. BMS	0.64	(0.44, 0.93)	**0.64**	**(0.44, 0.93)**
Polymeric PES vs. BMS	0.26	(0.19, 0.36)	**0.27**	**(0.20, 0.37)**
Polymeric SES vs. BMS	0.17	(0.12, 0.24)	**0.20**	**(0.13, 0.30)**

Polymeric EES vs. AES	0.12	(0.01, 0.73)	0.14	(0.02, 1.04)*
MES vs. AES	0.36	(0.10, 1.34)	0.36	(0.09, 1.44)
Apolymeric PES vs. AES	0.25	(0.11, 0.54)	0.25	(0.11, 1.57)
Polymeric PES vs. AES	0.10	(0.05, 0.21)	0.10	(0.04, 0.23)
Polymeric SES vs. AES	0.07	(0.03, 0.14)	0.07	(0.03, 0.18)

MES vs. Polymeric EES	3.00	(0.40, 30.08)	2.45	(0.28, 21.48)***
Apolymeric PES vs. Polymeric EES	2.06	(0.37, 16.49)	1.72	(0.26, 11.08)***
Polymeric PES vs. Polymeric EES	0.85	(0.15, 6.79)	0.72	(0.11, 4.61)***
Polymeric SES vs. Polymeric EES	0.56	(0.10, 4.53)	0.54	(0.08, 3.50)***

Apolymeric PES vs. MES	0.69	(0.21, 2.16))	0.70	(0.20, 2.42)
Polymeric PES vs. MES	0.28	(0.09, 0.87)	0.29	(0.08, 1.00)
Polymeric SES vs. MES	0.19	(0.06, 0.58	0.21	(0.06, 0.77)

Polymeric PES vs. Apolymeric PES	0.41	(0.25, 0.67)	0.42	(0.26, 0.68)
Polymeric SES vs. Apolymeric PES	0.27	(0.16, 0.45)	0.31	(0.17, 0.55)

Polymeric SES vs. Polymeric PES	0.66	(0.41, 1.05)	0.74	(0.43, 1.25)

Bolded text denotes head-to-head meta-analysis evaluations. * Mixed treatment method identifies a significant effect, adjusted indirect comparison does not, adjusted indirect comparison identifies significant effect, mixed treatment comparison does not. ** Direction of effect differs between approaches, ***Important effect size differences.

#### Analysis 2. NSAIDS for acute pain[19]

We evaluated the effects of 7 different interventions for acute pain from 29 trials that included 58 trial arms, for a possible 21 comparisons. See Figure 2 and Table 2. We found no important distinctions between the adjusted indirect comparison and mixed treatment comparison approaches.

**Table 2:** NSAIDS for acute pain[19].

**Treatment Comparison**	**Mixed Treatment Comparison**		**Adjusted Indirect Comparison**	
	
	**Odds Ratio**	**95% Credible Interval**	**Odds Ratio**	**95% Confidence Interval**
Ketoprofen vs. Placebo	6.55	(4.35, 9.95)	**6.06**	**(4.07, 9.04)**
Ibuprofen vs. Placebo	2.95	(1.92, 4.57)	**2.70**	**(1.78, 4.09)**
Felbinac vs. Placebo	3.02	(2.01, 4.58)	**2.91**	**(1.94, 4.39)**
Piroxicam vs. Placebo	2.75	(1.86, 4.08)	**2.65**	**(1.80, 3.90)**
Indomethacin vs. Placebo	1.60	(0.99, 2.62)	**1.58**	**(0.97, 2.57)**
Other NSAID vs. Placebo	3.74	(2.73, 5.13)	**3.31**	**(2.46, 4.45**

Ibuprofen vs. Ketoprofen	0.45	(0.25, 0.81)	0.44	(0.25, 0.79)
Felbinac vs. Ketoprofen	0.46	(0.26, 0.83)	0.48	(0.27, 0.84)
Piroxicam vs. Ketoprofen	0.42	(0.24, 0.74)	0.43	(0.25, 0.76)
Indomethacin vs. Ketoprofen	0.24	(0.13, 0.46)	0.26	(0.13, 0.48)
Other NSAID vs. Ketoprofen	0.57	(0.34, 0.95)	0.54	(0.33, 0.89)

Felbinac vs. Ibuprofen	1.02	(0.56, 1.86)	1.07	(0.60, 1.92)
Piroxicam vs. Ibuprofen	0.93	(0.52, 1.67)	0.98	(0.55, 1.73)
Indomethacin vs. Ibuprofen	0.54	(0.29, 1.04)	0.58	(0.30, 1.11)
Other NSAID vs. Ibuprofen	1.27	(0.74, 2.15)	1.22	(0.73, 2.04)

Piroxicam vs. Felbinac	0.91	(0.52, 1.60)	0.91	(0.51, 1.59)
Indomethacin vs. Felbinac	0.53	(0.28, 1.01)	0.54	(0.28, 1.02)
Other NSAID vs. Felbinac	1.24	(0.74, 2.08)	1.13	(0.68, 1.88)

Indomethacin vs. Piroxicam	0.58	(0.31, 1.09)	0.59	(0.31, 1.11)
Other NSAID vs. Piroxicam	1.36	(0.82, 2.25)	1.24	(0.76, 2.03)

Other NSAID vs. Indomethacin	2.33	(1.31, 4.15)	2.09	(1.18, 3.70)

Bolded text denotes head-to-head meta-analysis evaluations. * Mixed treatment method identifies a significant effect, adjusted indirect comparison does not, adjusted indirect comparison identifies significant effect, mixed treatment comparison does not. ** Direction of effect differs between approaches, ***Important effect size differences.

#### Analysis 3. Statins for the primary prevention of cardiovascular mortality[17]

We evaluated the role of 4 statin interventions compared to placebo/standard care for the prevention of cardiovascular mortality in primary prevention of cardiovascular disease populations. See Figure 3 and Table 3. There were 18 trials included, from 38 arms, allowing for a possible 10 comparisons. We found no major discrepancies between the two comparative approaches.

**Table 3:** Statins for the prevention of cardiovascular mortality[17].

**Treatment Comparison**	**Mixed Treatment Comparison**		**Adjusted Indirect Comparison**	
	
	**Odds Ratio**	**95% Credible Interval**	**Odds Ratio**	**95% Confidence Intervals**
Atorvastatin vs. Placebo	0.88	(0.69, 1.11)	**0.88**	**(0.70, 1.12)**
Fluvastatin vs. Placebo	0.77	(0.53, 1.11)	**0.77**	**(0.53, 1.11)**
Pravastatin vs. Placebo	0.91	(0.80, 1.03)	**0.91**	**(0.81, 1.02)**
Lovastatin vs. Placebo	0.67	(0.35, 1.24)	**0.55**	**(0.31, 0.99)**

Fluvastatin vs. Atorvastatin	0.87	(0.56, 1.35)	0.87	(0.56, 1.35)
Pravastatin vs. Atorvastatin	1.03	(0.79, 1.35)	1.03	(0.79, 1.33)
Lovastatin vs. Atorvastatin	0.76	(0.38, 1.47)	0.62	(0.33, 1.15)

Pravastatin vs. Fluvastatin	1.18	(0.80, 1.75)	1.18	(0.79, 1.74)
Lovastatin vs. Fluvastatin	0.87	(0.41, 1.80)	0.71	(0.36, 1.41)

Lovastatin vs. Pravastatin	0.74	(0.39, 1.38)	0.60	(0.33, 1.08)

Bolded text denotes head-to-head meta-analysis evaluations. * Mixed treatment method identifies a significant effect, adjusted indirect comparison does not, adjusted indirect comparison identifies significant effect, mixed treatment comparison does not. ** Direction of effect differs between approaches, ***Important effect size differences.

#### Analysis 4. Topical treatment for treatment of ear discharge at 1 and 2 weeks [21]

We evaluated the role of topical antibiotics for the prevention of ear discharge for patients with eardrum perforations using 18 2-arm randomised trials comparing 4 different treatments. Figure 4 displays the network of evidence available from these trials. The results of the 2 pair-wise treatment comparisons performed via the adjusted indirect comparison approach and 6 pair-wise treatment comparisons performed via the mixed treatment comparison approach are shown in Table 4. In one circumstance, the mixed treatment comparison approach found a statistically significant difference between the effects of two treatments, when the adjusted indirect comparison approach did not.

**Table 4:** Topical treatment for treatment of ear discharge at 1 and 2 weeks[21].

**Treatment Comparison**	**Mixed Treatment Comparison**		**Adjusted Indirect Comparison**	
	
	**Odds Ratio**	**95% Credible Interval**	**Odds Ratio**	**95% Confidence Interval**
Topical Quinolone Antibiotic vs. No Treatment	0.13	(0.06, 0.24)	**0.08**	**(0.01, 0.51)**
Topical Non-Quinolone Antibiotic vs. No Treatment	0.21	(0.10, 0.44)	0.28	(0.09, 0.52)
Topical Antiseptic vs. No Treatment	0.71	(0.32, 1.55)	0.61	(0.22, 1.22)

Topical Non-Quinolone Antibiotic vs. Topical Quinolone Antibiotic	1.67	(1.17, 2.31)	**1.62**	**(0.92, 2.85)**
Topical Antiseptic vs. Topical Quinolone Antibiotic	5.64	(3.70, 8.70)	**4.31**	**(1.34, 13.90)**

Topical Antiseptic vs. Topical Non-Quinolone Antibiotic	3.37	(2.25, 5.03)	**3.02**	(0.74, 12.29)*

Bolded text denotes head-to-head meta-analysis evaluations. * Mixed treatment method identifies a significant effect, adjusted indirect comparison does not, adjusted indirect comparison identifies significant effect, mixed treatment comparison does not. ** Direction of effect differs between approaches, ***Important effect size differences.

#### Analysis 5. Antifungal agents for preventing mortality in solid organ transplant recipients[20]

We evaluated the role of antifungal agents for preventing mortality in solid organ transplant recipients on the basis of 10 2-arm randomised trials comparing 5 different treatments. The network of evidence for these trials is shown in Figure 5. The results for the 5 possible pair-wise treatment comparisons using the adjusted indirect comparison approach and 10 comparisons using the mixed treatment comparison are shown in Table 5. In a single case, the mixed treatment comparison approach found a different direction of effect than the adjusted indirect comparison approach. The estimated ORs and associated uncertainty intervals produced by the two approaches were importantly different for three pair-wise treatment comparisons.

**Table 5:** Antifungal agents for preventing mortality in solid organ transplant recipients[20].

**Treatment Comparison**	**Mixed Treatment Comparison**		**Adjusted Indirect Comparison**	
	
	**Odds Ratio**	**95% Credible Interval**	**Odds Ratio**	**95% Confidence Interval**
Fluconazole vs. Control	0.81	(0.48, 1.37)	**0.94**	**(0.54, 1.63)**
Itraconazole vs. Control	0.90	(0.41, 1.99)	**0.49**	**(0.15, 1.56)**
Liposomal Amphotericin B vs. Control	0.50	(0.09, 2.33)	**0.54**	**(0.10, 2.52)**
Ketoconazole vs. Control	1.83	(0.38, 8.93)	**1.66**	**(0.41, 6.66)**

Intraconazole vs. Fluconazole	1.12	(0.52, 2.41)	**1.69**	**(0.58, 5.33)**
Liposomal Amphotericin B vs. Fluconazole	0.62	(0.10, 3.17)	0.57	(0.09, 3.39)
	
Ketoconazole vs. Fluconazole	2.27	(0.43, 12.09)	1.76	(0.39, 7.94)***

Liposomal Amphotericin B vs. Itraconazole	0.55	(0.09, 3.11)	1.10	(0.14, 8.65)**, ***
Ketoconazole vs. Itraconazole	2.03	(0.35, 11.87)	3.38	(0.54, 21.16)***

Ketoconazole vs. Liposomal Amphotericine B	3.68	(0.41, 35.30)	3.07	(0.34, 27.48)***

Bolded text denotes head-to-head meta-analysis evaluations. * Mixed treatment method identifies a significant effect, adjusted indirect comparison does not, adjusted indirect comparison identifies significant effect, mixed treatment comparison does not. ** Direction of effect differs between approaches, ***Important effect size differences.

#### Analysis 6. Prophylactic treatments against pneumocystis carinii pneumonia and toxoplasma encephalitis in HIV-infected patients[4]

We evaluated 4 different interventions from 22 trials with 44 trial arms, allowing a possible 6 comparisons. See Figure 6 and Table 6. In this example, the adjusted indirect comparison was only required for one comparison but differed importantly from the mixed treatment method.

**Table 6:** Prophylactic treatments against pneumocystis carinii pneumonia and toxoplasma encephalitis in HIV-infected patients[4].

**Treatment Comparison**	**Mixed Treatment Comparison**		**Adjusted Indirect Comparison**	
	
	**Odds Ratio**	**95% Credible Interval**	**Odds Ratio**	**95% Confidence Interval**
AP vs. TMP-SMX	2.68	(1.90, 3.81)	**3.19**	**(2.02, 5.03)**
D vs. TMP-SMX	1.38	(0.94, 2.04)	**0.92**	**(0.58, 1.46)**
D/P vs. TMP-SMX	3.02	(1.92, 4.79)	**3.22**	**(1.70, 6.10)**

D vs. AP	0.52	(0.34, 0.78)	**0.90**	**(0.53, 1.52)**
D/P vs. AP	1.13	(0.71, 1.80)	**0.86**	**(0.47, 1.57)**

D/P vs. D	2.19	(1.26, 3.82)	3.5	(1.59, 7.69)***

Bolded text denotes head-to-head meta-analysis evaluations. * Mixed treatment method identifies a significant effect, adjusted indirect comparison does not, adjusted indirect comparison identifies significant effect, mixed treatment comparison does not. ** Direction of effect differs between approaches, ***Important effect size differences.

#### Analysis 7. Antifungal agents for the prevention of mortality among patients with invasive candidemia

(Perri D, O'Regan C, Cooper C, Nachega JB, Wu P, Tleyjeh I, Philips P, Mills EJ: Antifungal treatment for systemtic candida infectons: A mixed treatment comparison meta-analysis. Unpublished.)

We evaluated the effectiveness of 8 different treatments from 19 trials, allowing 38 arms, for a possible 28 comparisons. See Figure 7 and Table 7. For 9 comparisons we were unable to conduct the adjusted indirect evaluation, as no suitable mutual comparator existed. The direction of effect differed between the two approaches in 4 studies. In one circumstance, the adjusted indirect approach found significant treatment effect while the mixed treatment method did not.

**Table 7:** Antifungal agents for the prevention of mortality among patients with invasive candidemia (Perri D, O'Regan C, Cooper C, Nachega JB, Wu P, Tleyjeh I, Philips P, Mills EJ: Antifungal treatment for systemtic candida infectons: A mixed treatment comparison meta-analysis. Unpublished)

**Treatment Comparison**	**Mixed Treatment Comparison**		**Adjusted Indirect Comparison**	
	
	**Odds Ratio**	**95% Credible Interval**	**Odds Ratio**	**95% Confidence Interval**
Caspofungin vs. Fluconazole	1.01	(0.60, 1.71)	0.85	(0.44, 1.64)**
Amphotericin B Deoxycholate vs. Fluconazole	1.26	(0.96, 1.65)	**1.31**	**(0.99, 1.74)**
Amphotericin B Liposomal vs. Fluconazole	1.20	(0.70, 2.06)	**-**	**-**
Voriconazole vs. Fluconazole	1.20	(0.75, 1.94)	0.58	(0.33, 0.99)*, ***
Micafungin vs. Fluconazole	1.22	(0.68, 2.16)	**-**	**-**
Anidulafungin vs. Fluconazole	0.64	(0.36, 1.14)	**0.65**	**(0.35, 1.19)**
Itraconazole vs. Fluconazole	0.89	(0.46, 1.69	0.54	(0.27, 1.05)

Amphotericin B Deoxycholate vs. Caspofungin	1.24	(0.79, 1.94)	**1.12**	**(0.62, 1.12)**
Amphotericin B Liposomal vs. Caspofungin	1.19	(0.90, 1.57)	**1.31**	**(0.90, 1.91)**
Voriconazole vs. Caspofungin	1.19	(0.75, 1.89)	0.67	(0.32, 1.43)**
Micafungin vs. Caspofungin	1.20	(0.89, 1.62)	**1.25**	**(0.84, 1.88)**
Anidulafungin vs. Caspofungin	0.64	(0.29, 1.38)	**-**	**-**
Itraconazole vs. Caspofungin	0.87	(0.41, 1.84	0.63	(0.27, 1.47)

Amphotericin B Liposomal vs. Amphotericin B Deoxycholate	0.96	(0.60, 1.53)	1.80	(0.86, 3.76)**
Voriconazole vs. Amphotericin B Deoxycholate	0.96	(0.65, 1.42)	**0.76**	**(0.48, 1.22)**
Micafungin vs. Amphotericin B Deoxycholate	0.97	(0.58, 1.61)	0.95	(0.55, 1.64)
Anidulafungin vs. Amphotericin B Deoxycholate	0.51	(0.27, 0.96)	0.49	(0.25, 0.97)
Itraconazole vs. Amphotericin B Deoxycholate	0.70	(0.39, 1.27)	**0.71**	**(0.39, 1.28)**

Voriconazole vs. Amphotericin B Liposomal	1.00	(0.64, 1.55)	**1.37**	**(0.77, 2.45)**
Micafungin vs. Amphotericin B Liposomal	1.01	(0.75, 1.35)	**0.96**	**(0.66, 1.40)**
Anidulafungin vs. Amphotericin B Liposomal	0.54	(0.24, 1.17)	**-**	**-**
Itraconazole vs. Amphotericin B Liposomal	0.74	(0.34, 1.57)	**-**	**-**

Micafungin vs. Voriconazole	1.01	(0.61, 1.67)	-	-
Anidulafungin vs. Voriconazole	0.54	(0.25, 1.13)	-	-
Itraconazole vs. Voriconazole	0.74	(0.36, 1.50)	0.93	(0.43, 1.98)

Anidulafungin vs. Micafungin	0.53	(0.24, 1.19)	-	-
Itraconazole vs. Micafungin	0.73	(0.33, 1.59)	-	-

Itraconazole vs. Anidulafungin	1.37	(0.58, 3.26)	-	-

Bolded text denotes head-to-head meta-analysis evaluations. * Mixed treatment method identifies a significant effect, adjusted indirect comparison does not, adjusted indirect comparison identifies significant effect, mixed treatment comparison does not. ** Direction of effect differs between approaches, ***Important effect size differences.

## Discussion

Our paper presents important evidence on the relative performance of the adjusted indirect comparison and mixed treatment comparison approaches to evaluating multiple health interventions in the absence of sufficient direct evidence.

For the 3 star-networks considered in this paper, we found that both approaches led to similar results, as they could use all the available information in the data. In general, some slight difference may exist between the results produced by the two approaches for this type of network since the adjusted indirect comparison approach uses (approximate) normal likelihood while the mixed treatment comparison approach uses (exact) binomial likelihood. If one chooses to ignore such a slight difference, the adjusted indirect comparison approach is easier to use for star-networks than the mixed treatment comparison approach.

For the 2 single-loop networks included in this paper, we found that the adjusted indirect comparison and mixed treatment comparison approached yielded comparable estimates of relative treatment effectiveness. However, the two approaches will be expected to yield different results for general single-loop networks, simply because the mixed treatment comparison approach is based on all available information in the data but the adjusted indirect comparison approach is not.

Finally, we found that both the adjusted indirect comparison and the mixed treatment comparison approach produced comparable estimates of relative treatment effectiveness for the two multi-loop networks considered in this paper. As pointed out by one of the referees during peer-review, in general, the adjusted indirect comparison approach may be difficult, if not impossible, to apply for this type of network. As an illustration, suppose we are interested in the indirect estimate for the OR of the pairwise comparison of treatments C and D in Figure 7, where there is no direct comparison between these two treatments. But, through the network of evidence, there are three ways to perform the adjusted indirect comparison of treatments C and D: (1) using comparisons C versus E and D versus E; (2) using comparisons C versus B and D versus B; (3) using comparisons C versus B, B versus F, and F versus D. Clearly, these comparisons will lead to different results. One possible way to deal with this problem is to apply the adjusted indirect comparison approach three times to these data sets respectively and then combine them together to get a pooled estimate. But, crucially, these three routes to the estimate of the OR of the pair-wise comparison of treatments C and D are not in this case statistically independent. As a result, the resulting estimates cannot be pooled by a simple weighted average. The mixed treatment comparisons approach, however, will combine this information simultaneously and produce a coherent set of estimates for all the treatment contrasts, based on all the data.

The adjusted indirect comparison approach may be preferred for star-networks, as it is typically easier to implement than the mixed treatment comparison approach and provides similar results. For single-loop networks, one could use either approach, though the results produced by the two approaches might generally be different, reflecting the fact that the mixed treatment comparison approach relies on all of the information available in the data but the adjusted indirect comparison approach does not. For multi-loop networks, it might be difficult, if not impossible, to implement the adjusted indirect comparison approach in some situations, rendering the mixed treatment comparison as the preferred choice for this type of network.

There are strengths and limitations that should be considered when interpreting this manuscript. Strengths include our extensive searching of systematic reviews and inclusion of unpublished systematic reviews. It is possible that we missed systematic reviews that may have met our inclusion criteria, however our searches were extensive, were supplemented with others' systematic reviews.[2,13] and were conducted in duplicate to minimize bias. We applied the fixed effects method for both the adjusted indirect comparison and mixed treatment comparison approaches. Our goodness of fit checks indicated that the fixed effects mixed treatment comparison approach was sensible for nearly all of the seven systematic reviews. Further sensitivity analyses performed for this approach confirmed the robustness of the overall conclusions to the exclusion of discrepant trials. It is possible that we would have found slight differences if we had employed the random effects method. More often than not however, these methods yield comparable estimates of relative treatment effects.[18] Some have argued that the fixed effects method should now be preferred over a random effects method as it places a greater weight on larger studies, thus studies may have reduced bias.[23] Finally, we were unable to compare the adjusted indirect comparison approach with the head-to-head evaluations as, in this set of systematic reviews, there was an insufficient number of trials with more than one comparator.

Salanti and others have discussed the merits and challenges of the mixed treatment approach.[12,23,24] The mixed treatment comparison is a resource intensive approach to conducting analyses as it requires knowledge of Bayesian principles and working abilities with WinBUGS, a somewhat user-unfriendly software for those unfamiliar with it. However, the mixed treatment approach also provides interesting additional information that may be useful to some readers. Additional information includes probabilities of a ranking order of the effectiveness of interventions. For the sake of clarity, we haven't presented the probabilities associated with each analysis. Probabilities may be difficult to interpret though, particularly when there are not clear differences amongst them. A further additional source of information is that this analysis provides indirect comparisons without requiring a mutual comparator, a possible strength over the adjusted indirect approach. However, we cannot know whether this estimate is reliable or similar to an adjusted indirect approach until further trials become available. Finally, some have argued that the mixed treatment comparison is a 'black-box,' as it may be difficult or impossible to determine where an analysis has gone incorrectly.[25] Future validations of the analytic manner performed in this manuscript may yield insights into the transparency of this method. Finally, no reporting guidelines exist for the mixed treatment approach. A step forward may be the development of minimum reporting criteria for this approach.[11,12]

For less complex analyses, such as star-shaped networks, the adjusted indirect comparison may be easier for meta-analysts to apply in their general practice. One of us (GG) was involved in the development of this approach.[4] The adjusted indirect comparison is limited in more complex evaluations, as compared to the mixed treatment comparison, as it requires the utilization of a mutual comparator when performing indirect comparisons. However, as discussed above, the validity of indirect comparisons without mutual comparators that are performed via the mixed treatment comparison approach may be reasonably questioned. The adjusted indirect comparison approach requires the knowledge of standard meta-analysis techniques and working knowledge of programmable software such as R, S-Plus, Stata or SAS, so is arguably also resource intensive. A recent free download of a simple software may make this approach accessible for non-statisticians.[25,26]

There is also concern that both the adjusted indirect comparison and mixed treatment comparison approaches will have less power than the direct approach and may sometimes lead to indeterminate results, in the form of wide uncertainty intervals for relative intervention effects. Inferences based on such findings may therefore be limited. In addition, it is not clear yet how to interpret results that differ substantially between the two approaches. Finally, although the choice of approach may differ only marginally in treatment effect estimates, the impact of small differences may affect future analyses based on study findings, such as cost-effectiveness models. There is a clear need to evaluate whether one method may importantly impact cost-effectiveness projections over another.[11]

## Conclusion

In conclusion, both the mixed treatment comparison approach and the adjusted indirect comparison approach provide compelling inferences about the relative effectiveness of interventions. In less complex indirect comparisons, where a mutual comparator exists, the adjusted indirect comparison may be favourable due to its simplicity. In more complex models, the mixed treatment comparison appears to offer benefits for comparisons that other methods cannot.

## Abbreviations

AES: actinomycin-d-eluting **stent**; BMS: bare-metal stents, PES; EES: everolimus-eluting stent; MES: micophenolate-eluting stent; PES: paclitaxel-eluting stent; SES: sirolimus-eluting stent; RCT: randomized clinical trial; OR: odds ratio; CI: confidence interval; CrI: credible interval; D: Dapsone; D/P: dapsone/pyrimethamine; AP: aerosolized pentamidine; TMP-SMX: trimethoprim-sulfamethoxazole.

## Competing interests

None declared. COR has previously been employed by Pfizer Ltd. and is currently employed by Merck, Sharpe & Dohme (MSD) Ltd. MSD had no role in the development, execution or publication of the paper. EM has consulted to Pfizer Ltd. and received unrestricted research grants from Pfizer Ltd., GG has received unrestricted research grants from several for-profit companies. IG runs a statistical consulting firm.

## Authors' contributions

COR, EM, IG and GG were responsible for the study concept. COR, EM and OE were responsible for the study searches. COR, EM, EO, IG and GG were responsible for study extraction and analysis. COR, EM, IG and GG were responsible for study writing. COR, IG, EO, GG and EM approved the submitted manuscript.
